# The Long-Term Effect of Job Loss on Mortality

**DOI:** 10.1093/geroni/igaa057.3395

**Published:** 2020-12-16

**Authors:** Qian Song, Emily Lim

**Affiliations:** University of Massachusetts Boston, Boston, Massachusetts, United States

## Abstract

The associations between job loss and health were established. However, these associations were usually biased estimates of causality, as individuals with adverse health were self-selected into job loss. To address such selectivity, we take advantage of the case of massive layoffs of State-Owned Enterprise (SOE) workers in urban China, which was driven by policy change rather than individual characteristics and mimicked a quasi-experiment. We employ China Health and Nutrition Survey (1991-2015) that span more than two decades and survival analysis to analyze the effect of job loss on individual mortality. Our analyses with 3,494 SOE workers show that over the past twenty years, job loss has accelerated mortality (hazard ratio = 1.50; p<0.05) for SOE workers in urban China. Further, these accelerated moralities occurred mostly for workers who lost their jobs between 30-39 (hazard ratio = 2.52, p<0.05) and 40-50 (hazard ratio = 1.77, p<0.1) years old, highlighting the adverse consequences of job loss for the young and middle-aged workers. Our analyses established the causality of job loss and mortality over an extended time, and identified the most vulnerable groups to job loss for policies to target to in an effort to improving the well-being of workers later in life.

